# Charting the course of asymmetric cell division in maize: The crucial role of OPAQUE1 in guiding the phragmoplast

**DOI:** 10.1093/plcell/koad104

**Published:** 2023-04-10

**Authors:** Arpita Yadav

**Affiliations:** Assistant Features Editor, The Plant Cell, American Society of Plant Biologists; Biology Department, University of Massachusetts, Amherst, MA 01003, USA

Stomata are specialized pores located on the surface of leaves and other plant organs that play an important role in gas exchange, water loss regulation, and carbon dioxide uptake for photosynthesis. Stomata development in maize and other grasses occurs through a series of asymmetric and symmetric cell divisions.

Formation of a grass stomatal complex requires 3 divisions: 1) asymmetric division of a protodermal cell to form a guard mother cell (GMC) and an interstomatal cell, 2) asymmetric division of 2 subsidiary mother cells (SMCs) flanking the GMC to form subsidiary cells, and 3) symmetric but oriented division of GMC to form 2 guard cells. Subsidiary cells are intimately associated with guard cells, creating a 4-celled stomatal complex that orchestrates efficient gas exchange. The asymmetric division of SMCs provides an interesting model to study cell division, facilitated by the presence of morphologically different daughter cells and the availability of clear polarization markers. Mutants that incorrectly execute stomatal divisions are classified depending on which phase of asymmetric division is defective: cell polarization, division plane establishment, division plane maintenance, or cytokinesis. For example, the *brick1* (*brk1*), *brk2*, and *brk3* mutants are defective in cell polarization.

The microtubule-binding protein TANGLED1 (TAN1) is a crucial division site marker from prophase to telophase (Smith et al. 1996). TAN1 interacting partners POK1 (PHRAGMOPLAST ORIENTING KINESIN1) and POK2 are kinesins that are localized to the phragmoplast and the cortical division site (Lipka et al. 2014). The phragmoplast acts as a scaffold during asymmetric cell division, directing the creation of the cell plate and new cell wall (Smertenko 2018). This is significant because the cell plate and new cell wall must be created in a precise manner to produce 2 daughter cells with distinct fates. The phragmoplast is made of membranes, microtubules, actin, and other proteins that form a disc at the center, which circumferentially expands until it meets the cortical division site, marked by TAN1, POK1, and others. After phragmoplast expansion is complete, the cell plate fuses with the parental plasma membrane and existing cell wall. Phragmoplast expansion occurs in distinct stages encompassing the integrity of phragmoplast and early compared with late phragmoplast guidance, but the precise mechanisms and protein-protein interactions that promote correct phragmoplast guidance through theses stages remain elusive.

In this issue, **Qiong Nan and colleagues (Nan et al. 2023**) aimed to learn how plant cells correctly execute the asymmetric division of maize (*Zea mays*) SMCs. In previous research, it was shown that actin motors and myosins are key components of SMC division during polarization, establishing division planes and/or cytokinesis. The authors investigated asymmetric divisions in maize stomata using the previously identified OPAQUE ENDOSPERM 1 (O1) protein, a myosin XI protein necessary for normal endoplasmic reticulum and protein body morphology during seed development **(Wang et al. 2012**). The author’s observations in the *o1* mutant showed the presence of aberrant subsidiary cells and aborted GMCs, which suggested a role for O1 in the formative asymmetrical divisions. To substantiate the phenotype, they looked at the cell walls, plasmodesmata, and cell plates of dividing stomatal cells and found that the GMCs of *o1* mutants had abnormal division planes.

Next, they sought to determine the O1 function in the division plane more precisely, for example, whether in cell polarization division plane establishment, maintenance, or cytokinesis. Importantly, O1 was found to localize to and play a role in phragmoplast guidance during cytokinesis (Figure). Specifically, the late-stage phragmoplast is abnormal in *o1* mutant SMCs. Interestingly, the defects observed in the *o1* mutants were similar to the previously identified *discordia* (*dcd1* and *dcd2*) class of mutants that display altered post-polarization defects in SMC division (Gallagher and Smith 1999). However, the *dcd2* gene had not been identified to date. Here the authors reveal that *dcd2* is allelic to *o1*.

**Figure.**
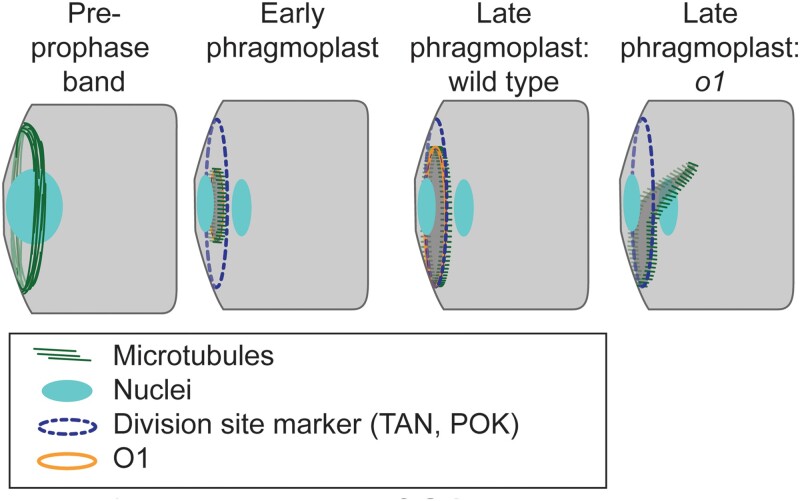
Role of O1 in phragmoplast guidance. The pre-prophase band marks the inicipient cortical division site, followed by markers for the division site such as TAN1 and POK proteins also present in the phragmoplast. Actin filaments and myosin VIII direct the phragmoplast to the division site in wild-type cells (Wu and Bezanilla 2014). The phragmoplast keeps growing when it encounters the cortex, and contacts are maintained by microtubules (Bellinger et al. 2021). POK, TAN, and O1 act as mediators as the cell plate fuses with the parental plasma membrane in the wild type along the established division site. In *o1* mutants, the phragmoplast becomes misguided following initial contact, leading to aberrant division planes. Adapted from Nan et al. (2023), Figure 9.

To learn more about how O1 influences phragmoplast guidance, the authors used co-immunoprecipitation and mass spectrometry to identify proteins associated with O1. Notably, in addition to the identification of actin-associated proteins, the authors discovered the microtubule-associated kinesins KIN12C, KIN12D, and KIN12E as putative O1 interactors. These 3 kinesins are related to Arabidopsis POK1, POK2, and KIN12E, which mark the cortical division site during cell division and interact with maize TAN1. Finally, because POK proteins are division site markers, the authors sought to examine if the defects in late-stage phragmoplast guidance in the *o1* mutant were due to defects in division site maintenance. However, localization of TAN1, a marker of the division site, was not altered in dividing *o1* mutant SMC and GMC progenitor cells, indicating that O1 is essential for phragmoplast guidance to the specified division sites during cytokinesis.
